# Arnold–Chiari Malformations in Pediatric Patients After Fetal Surgery for Meningomyelocele

**DOI:** 10.3390/jcm13226721

**Published:** 2024-11-08

**Authors:** Miroslava Kohútková, František Horn

**Affiliations:** Department of Paediatric Surgery, Faculty of Medicine, Comenius University of Bratislava, 833 40 Bratislava, Slovakia

**Keywords:** pediatric neurosurgery, neural tube defects, in utero open fetal meningomyelocele repair, Arnold–Chiari malformations, decompression of craniocervical junction, intracranial hypertension, hydrocephalus

## Abstract

(1) **Background**: Fetal surgery for meningomyelocele (MMC) should reduce the occurrence of Arnold–Chiari malformations, hydrocephalus, the associated need for craniocervical decompression, and the need for cerebrospinal fluid shunt insertion. Fetal surgery should improve ambulatory status. (2) **Methods**: We used retrospective analysis of the documentation and descriptive statistics to summarize the clinical data and measured MRI parameters. The neurosurgical results are presented as the frequency of findings in percentages and compared with the results of the Management of Myelomeningocele Study (MOMS). (3) **Results**: A total of eight patients who underwent prenatal correction of MMC between 2016 and 2020 participated. MRI detected Chiari II malformation in all patients during prenatal imaging and in 87.5% of the patients at the age of 12 months. Craniocervical decompression was used in 25% of the patients. Shunt-dependent hydrocephalus occurred in 50% of the cases. In 87.5% of the cases, the functional level exceeded the expected ambulatory status. (4) **Conclusions**: We present the clinical status of our patients. Analysis of the complete cohort confirmed that prenatal surgery is associated with a reduced occurrence of Chiari malformations and reduced associated occurrence of hydrocephalus. Specific lesion levels are not associated with the need for craniocervical decompression. The results of our study are valuable in prenatal counseling and important for treatment planning.

## 1. Introduction

Neural tube defects (NTDs) are congenital anomalies. This spectrum includes open or closed cranial and spinal defects. The most appropriate division of NTDs is the division, according to location, into a group of cranial and a group of caudal defects. The given groups are then divided according to their coverage into open and closed types. In the open type, the nerve tissue is exposed to the outside environment, or it may be covered by a thin membrane so it is visible. Spina bifida aperta, or open spinal dysraphism, represents several malformations, such as myelomeningocele. Unlike them, when they are closed, the nerve tissue is covered by skin. Open forms are associated with neurological deficits and hydrocephalus. Closed forms do not necessarily have such an association, but a neurological deficit or hydrocephalus can develop over time. The most common and the most severe open form is myelomeningocele [1,2,3,4].

The major causes of stillbirth, neonatal, and infant death are neural tube defects. A total of 0.5–2 per 1000 pregnancies worldwide are affected by NTDs. NTDs are the reason for significant lifelong handicaps [1]. 

Nowadays, the occurrence of NTDs, especially MMC, has a decreasing tendency thanks to folate acid supplementation [2,3,4,5,6].

Neural tube defects occur at the beginning of fetal development during the first weeks of pregnancy. The emergence of the most serious forms can be dated between the third and fourth weeks of embryonic development. At the end of the third gestational week, the neural tube begins to close, and this process ends around the 27th day of fetal development. The fetus is particularly susceptible to teratogens in the period between the third and sixth weeks of pregnancy. As a result of the influence of genetic predisposition, undesirable factors, or, on the contrary, the lack of the necessary ones, results in the emergence of one or more vertebral arches, through which herniation of the spinal cord structures occurs [1,3,5,6]. The etiology of NTDs is multifactorial. Environmental factors are, for example, chemical teratogens, pesticides, organic solvents, or exposure to radiation. There can be a genetic predisposition in origin, but most cases are sporadic, with non-genetic patterns. 

The maternal factors include an inappropriate diet with low nutrition, low folic acid supplementation, caffeine and alcohol consumption, smoking, the use of anticonvulsants, maternal diabetes, obesity, hyperthermia, or anxiety, which can cause NTDs, too [3,4,5,6].

The two most common conditions associated with the brain in MMC are hydrocephalus and Arnold–Chiari II malformations. The majority of patients with MMC develop severe Chiari II malformation. This malformation is the extrusion of the cerebellar tonsils, vermis, and the fourth ventricle through the foramen magnum. Because of that, the medulla oblongata and the spinal cord are compressed [3,7,8].

The presence of a Chiari II malformation has an impact on the development of hydrocephalus and affects the neurological motor condition of the patient, too. This condition determines the outcome for the survival and independence of children. Patients usually have sensory and motor neurologic deficits, bowel dysfunction, and urinary dysfunction. The reason is described by the hypothesis of two interventions. This hypothesis, named the “two-hit hypothesis,” describes the exposure of the neural tissues to amniotic fluid in the surrounding environment. Secondary traumatic damage occurs due to amniotic fluid [7,8,9]. Fetal surgery tries to prevent the described second damage to the nervous tissue. The unified theory of McLone and Dias and McLone and Knepper is generally accepted as etiology. In the presence of a congenital lumbosacral open defect, there is a constant outflow of cerebrospinal fluid, which creates a pressure difference between the spinal and cranial spaces. The intracranial hypotension causes the descent of the cerebellar structures [4,9]. Early waterproof closure of the defect in utero aims to reverse the herniation of the hindbrain, protect the nerve tissues, prevent the leakage of cerebrospinal fluid, and preserve the neurological function of the spinal cord [10]. Based on the described rationale for in utero MMC repair, we decided to investigate the incidence of Chiari II malformation in our patients after fetal surgery and to evaluate the dependence between the occurrence of Chiari II malformation, hydrocephalus, and meningomyelocele. In this report, the patient’s motor outcomes are presented. We assessed the movement abilities of individual patients depending on the presence of Chiari II malformations. 

It is known that the motor functions of patients depend on the anatomical levels of lesions, too. In practice, it is possible to observe that the functional neurological disability is often shifted by the level of two or more vertebrae below the anatomical level [11]. 

Postnatal MRI is the gold standard for determining the expected functional level of patients with MMC, but fetal MRI also achieves similar results. Both examinations have a good predictive value for the long-term health status of patients with MMC. Vonzun, in 2022, tried to determine the anatomical level of the neural lesion using neuroimaging. The result was that it was not possible to accurately determine the anatomical level of the lesion by MRI or ultrasound. There will always be a one-level margin of error towards higher levels. These findings are important for prenatal counseling [12,13].

New studies suggest that there are more factors affecting the patient´s motor condition. One of them is the morphological configuration of the corpus callosum. According to the results of these studies, the corpus callosum is an important element in maintaining walking symmetry. The physiological position and shape of the corpus callosum and vermis are usually associated with better postnatal motor development of the child than what was predicted prenatally based on the lesion present. On MR images, a thorough study of the configuration of the corpus callosum and the localization of the vermis is necessary. These findings are very important in prenatal counseling [3,7].

An up-to-date, evidence-based review of the neurosurgical literature reveals that clinical outcomes following the fetal operations for MMC are favorable, but prenatal surgery for MMC carries different benefits and risks compared to traditional postnatal surgery. One of the most important studies related to fetal surgery is called the Management of Myelomeningocele Study (MOMS), and it is a randomized controlled trial. MOMS started in 2011. Nowadays, this study continues as MOMS3. The study was created for the purpose of comparing prenatal and postnatal repair of MMC. Before MOMS, the standard surgical procedure was postnatal repair of the MMC. The prenatal standardized approach included a maternal laparotomy, a stapled hysterotomy, dissection of the neural placode from surrounding tissues, primary closure of the dura, and primary closure of the fetal skin. The researchers found that prenatal repair of the MMC reversed hindbrain herniation and reduced the occurrence of shunt-dependent hydrocephalus. Mental development and motor function were better. The study was terminated early because of the benefits of prenatal repair. Nowadays, prenatal repair of the MMC as open fetal repair with watertight closure of the dura and closure of the overlying skin has become one of the treatment options. Prenatal repair can reduce the morbidity of NTD, but it is also associated with higher rates of obstetrical complications. These complications include oligohydramnios, chorioamniotic (CA) membrane separation, placental abruption, premature rupture of membranes (PROM), preterm delivery, and uterine scar dehiscence, and have a higher risk of occurrence compared to postnatal interventions [9,14,15,16,17,18].

## 2. Materials and Methods

The Medline (using the PubMed interface), Web of Science, and Scopus databases were searched for publication using the keywords fetal surgery of meningomyelocele. Works published from 1999 to 2024 were searched. Only relevant studies directly related to the fetal repair of the MMC were considered.

This study was carried out on the basis of a retrospective analysis of the documentation of patients after prenatal correction of the MMC, which was kept in the database of the Neurosurgery Department of the National Institute of Children’s Diseases in Bratislava. All consecutive patients undergoing prenatal open NTD repair between 2016 and 2020 at our institution were identified retrospectively through the hospital coding system. This study encompassed a cohort of 8 pediatric patients who underwent fetal MMC correction between 2016 and 2020. All patients were operated on at Kinderspital Zürich, Spina Bifida Zentrum. Postnatal operations were performed at the Neurosurgery Department of the National Institute of Children’s Diseases in Bratislava, Slovakia. The study period spanned from 2016 to 2024. 

NTDs are usually diagnosed during prenatal screening in the second trimester of pregnancy. The time interval from diagnosis to consultation is a few days. After prenatal consultation at our department, mothers are sent to Zurich for consultations about surgical indications. Fetal defect correction is performed between the 24th and 27th weeks of pregnancy. The birth is planned by section in the 37th week of pregnancy. Mothers and babies are monitored approximately 2 weeks after the sections. After monitoring in the neonatal care unit, the patient is discharged to home and outpatient care at the Kinderspital Zürich and the National Institute of Children´s Diseases in Bratislava. A clinical and administrative coordinator in Zürich and physicians in Bratislava are still available to the patient’s parents. The patient undergoes several professional examinations planned at the ages of 3, 6, 12, 18, and 24 months, followed by further examinations once per year until adulthood. Patient care is multidisciplinary and includes neurosurgical, neurological, pediatric, urological, orthopedic, and gastroenterology care. Descriptive statistics were used to summarize the clinical and demographic data, indicating the absolute and relative frequency. Obtained data and measured MRI parameters were evaluated by statistical analysis and are presented in percentages based on the frequency of findings.

All patients have prenatal and postnatal MRI images. MR imaging was used to determine the level of the lesion and the morphology of the corpus callosum. The need for craniocervical decompression was indicated based on clinical neurological examination and MRI. Insertion of a CSF shunt was indicated on the basis of a clinical examination, ultrasound through an open large fontanel, and CT scans. These indications were determined at the Slovak workplace. 

The functional level of lesion is widely used as an independent variable and determines the grading of the level of neurological function. The functional level of the lesion scale is determined according to muscle strength and according to which muscles are moving. 

The purpose of our retrospective study is to analyze the incidence and development in real time of Chiari malformations in patients with prenatally operated open neural tube defects. This study deals with the incidence and development of the Chiari II malformation in patients with prenatally operated open neural tube defects. In this study, we assessed the relationship between the functional level of the neural tube lesion and the need to undergo decompression of the craniocervical junction (CCJ). Furthermore, we evaluated the need to solve intracranial hypertension by endoscopic ventriculostomy of the third cerebral ventricle and implantation of a cerebrospinal shunt.

## 3. Results

Our cohort contains eight patients who underwent prenatal open neural tube defect repair. Five patients were girls, and three patients were boys. All patients had MMC detected prenatally at 24–25 weeks of gestation. All patients were delivered via cesarean section. The average gestation age at the time of treatment was 25 weeks, 1 day. The study spanned from 2016 to 2024. Patients underwent prenatal surgery between 2016 and 2020 at the Spinal Bifida Clinic at the Children’s Hospital in Zürich using an open method with a 3-layer closure of the defect. The mothers of patients underwent an open method of surgery, including maternal laparotomy followed by exteriorization of the uterus and a hysterotomy. After uterine exteriorization, MMC repair is performed in the same manner as in the postnatal approach. All mothers whom we sent for fetal surgery for MMC in the fetus to Zürich and who underwent the procedure had live births. Neither the mother nor the baby exited. Subsequent postnatal operations for hydrocephalus, Chiari malformation, and tethered spinal cord syndrome were performed in our workplace. Patients, after fetal surgery, were monitored in our department until adulthood. All patients had prenatal and repeated postnatal MR examinations.

Each patient in our study had a series of prenatal and postnatal MRIs of the brain and spinal cord. The Table 1 shows, that CM-II was present in 8/8 (100%) cases prenatally and postnatally after birth. A total of 7/8 (87.5%) patients manifested Chiari II malformation on MRI at the age of 12 months. The caudal descent of the cerebellar structures into the spinal canal beyond the foramen magnum (CM-II) was less than 3 mm in two cases, in the interval 3–5 mm in two cases, and more than 5 mm in four cases after birth. Discreet herniation less than 3 mm regressed in both patients at the age of 12 months. In 5/8 (62.5%) patients, the CM-II prenatally appeared at the same cervical spinal level as on postnatal MR images. In 1/8 (12.5%) patients, the vermis “ascended” postnatally by one segment. The most extensive herniation of the cerebellar tonsils was 14 mm, and this patient did not need decompression.

In this study, we present two cases with symptomatic Chiari II malformations who underwent decompression of the craniocervical junction. 

The first patient was a girl with borderline ventriculomegaly after prenatal correction of the MMC. The anatomical level of the lesion is L5, and her functional level is S1. At the age of 4, she had paroxysmal headaches for several months, sometimes repeatedly within a day, which usually lasted a few minutes and resolved spontaneously in the supine position. MRI showed a low position of the cerebellar tonsils and newly formed syringomyelia in the area of the craniocervical junction. The caudal descent or herniation of the cerebellar tonsils into the spinal canal was 3 mm on the right side and 7 mm on the left side beyond the foramen magnum. The patient underwent a decompressive laminectomy of the C1 vertebra and a partial laminectomy of the C2 vertebra, partial coagulation of the cerebellar tonsils, and duroplasty using a dura substitute. The patient did not have a headache after the operation, and she had no other problems. The postoperative neurological status has been stable. 

The second patient was a boy with three-chamber hydrocephalus and a Dandy–Walker variant of the posterior cranial fossa. The anatomical level of the lesion was L4-S2, and his functional level was S1. At the age of 6 months, the patient underwent endoscopic ventriculostomy of the third cerebral ventricle, and at the age of 9 months, a ventriculoperitoneal CSF shunt was implanted because of clinical symptoms of intracranial hypertension, verified on CT scans. At the control MRI at the age of 2.5 years, newly formed pressure manifestations of herniated cerebellar tonsils on the medulla oblongata were confirmed with discrete signal changes of the intramedullary basal type of edema, and displaced external and internal infratentorial cerebrospinal fluid spaces. The cerebellar tonsillar position at the age of 2.5 years below the foramen magnum was 6 mm on the right side and 5 mm on the left side. After birth, it was 8 mm on the right side and 6 mm on the left side. Regarding the neurological status, macrocrania was present as a mild hypertonic syndrome, with signs of mild paraparesis of the lower limbs and convergent strabismus. Subsequently, the patient underwent suboccipital decompression and C1 laminectomy. Postoperatively, the patient’s neurological status is stable, but the patient has a neurological urinary bladder and uses clean intermittent catheterization.

A total of 4/8 (50%) patients were diagnosed and treated for CM-II, along with hydrocephalus and myelomeningocele. We performed an endoscopic ventriculostomy of the third cerebral ventricle in one case, and we implanted a ventriculoperitoneal shunt in four cases. All shunts that were implanted were ventriculoperitoneal shunts with adjustable pressure valves. One patient, after endoscopic ventriculostomy of the third cerebral ventricle, needed reoperation after three months. Initial operations for hydrocephalus were performed in all four patients before the age of 12 months. All patients were indicated for surgery for clinical signs of intracranial hypertension. Two patients with shunt-dependent hydrocephalus have descent displacement of cerebellar structures less than 5 mm on MR scans, and two patients from this group have a herniation bigger than 5 mm.

## 4. Discussion

### 4.1. Morbidity and Complications in Wound After Fetal Surgery

Myelomeningocele (MMC) is the most common and severe form of spinal dysraphism. Fetal surgery for MMC is a delicate surgical procedure where fetal surgeons open the uterus and close open defects in the fetus’s back while the fetus is still in the mother´s womb. Because spinal cord damage is progressive during gestation, prenatal repair of the myelomeningocele may prevent further damage. Although there is demonstrated potential for fetal and pediatric benefits, there are significant maternal implications and complications that may occur acutely, postoperatively, for the duration of the pregnancy, and in subsequent pregnancies. Women with pregnancies complicated by fetal myelomeningocele who meet the established criteria for in utero repair should be counseled in a nondirective fashion regarding all management options, including the possibility of open maternal–fetal surgery. Maternal–fetal surgery for myelomeningocele repair should be offered only to carefully selected patients at facilities with an appropriate level of personnel and resources. The disadvantages of prenatal repair seem to be prematurity. All mothers whom we sent to Zürich met the conditions for fetal surgery, which they underwent. The inclusion and exclusion criteria were identical to the MOMS criteria. No mother or baby died during or after surgery. No mother or baby had life-threatening complications during or after the operation. In total, 2/8 (25%) patients had dehiscention in the operation wound after birth. One of these two patients had so big a defect that primary closure during prenatal surgery was impossible.

### 4.2. Chiari Malformations

The results from the MOMS trial show that the in utero repair decreased the frequency and severity of the Chiari II malformation. At 12 months of age, hindbrain herniation was present in 64% of the patients who underwent fetal MMC repair, compared to 96% of the patients who under postnatal repair [16].

The occurrence of CM-II in our study was 100% after birth and 75% at 12 months of age. Therefore, our results are not as favorable as those in the MOMS study.

In case of increased pressure on the cerebellum and on the structures of the craniocervical junction, surgery is required to restore the normal flow of cerebrospinal fluid. Many children with Chiari malformations have hydrocephalus. These children need a CSF shunt shortly after birth. First, it is necessary to evaluate whether there is hydrocephalus with pressure manifestations on the surrounding structures. In the case of the presence of both, the hydrocephalus should be addressed first. If the child has a shunt, its functionality should be checked first. When hydrocephalus is ruled out as the cause of the patient´s difficulties, Chiari malformation is resolved. The most effective treatment method is surgical decompression of the craniocervical junction. This operation includes suboccipital craniectomy, cervical laminectomy, and durotomy with or without dural augmentation. Chiari II malformation is characterized by herniation of the cerebellar tonsils through the foramen magnum to the low cervical levels, where they cause obstruction of the flow of cerebrospinal fluid. The operation restores normal circulation of the cerebrospinal fluid. Suboccipital craniectomy is usually small and ends at the point where herniation of the tonsils ends. A surgeon must pay attention to the localization of the torcular, which is often displaced more caudally than usual, closer to the foramen magnum. The extent of the suboccipital craniectomy is usually small. This operation is indicated in cases with clinical manifestation. The clinical presentation of Chiari II can be different. This presentation includes spinal symptoms because of the meningomyelocele, tethered cord syndrome, or syringomyelia, as well as symptoms of secondary hydrocephalus, brainstem symptoms, or cranial nerve dysfunction [4,7,19,20,21,22].

There is no indication for prophylactic decompressive surgery. Surgery is indicated if the patient has any clinical signs of malformation. Another indication is a clinically manifested syrinx caused by a malformation [4].

Treatment focuses on the decompression of the tonsillar concomitantly brain stem and restoration of a normal CSF flow. Surgery still remains the most effective method in achieving these goals and alleviating symptoms [4]. 

Patients in this study benefited greatly from the decompression of the craniocervical junction.

The motor skills of patients depend on the level of their MMC lesion. Table 2 shows the anatomical levels of the lesions of individual patients based on prenatal MR. Two patients, who are mobile only with the help of a wheelchair, have lesions at levels T12-S2 and T11-L1. The fixation of the spinal cord to the surrounding tissues was at the levels of T12-L1 and L3. An interesting fact is that patients whose mobility status can be considered the most difficult did not have the most severe MMC. The patient with the most extensive MMC had a defect from vertebrae T12 to S5, and he is currently mobile with the help of another person.

It is not rare that patients with CM-II and MMC have an atypical corpus callosum (CC) on their MR scans. Now, we know that the CC can affect a patient´s mobility. One of the purposes of this study was to investigate whether there is an association between worse mobility and atypical morphology of the CC. We used a classification system of the morphology of the CC based on Edwards et al. There are four classes. First is the normal, inconspicuous form. The second is a thinned hydrocephalic or hypoplastic form. Third is dysgenetic, a small form with abnormally shaped or missing areas, especially in the region of the splenium and rostrum. The last one is a combination of groups 2 and 3, so it is a combination of hydrocephalic and dysgenetic [7,21,22].

On the MR images of our patients, the normal morphology of the corpus callosum was confirmed in three cases (37.5%), and in five cases (62.5%), it was dysgenesis. Of these three patients with a physiological picture of the corpus callosum, two are able to walk independently with the help of orthopedic orthoses of the lower limbs, and one needs the help of a second person. The same mobility ability was also found among patients with dysgenesis of the corpus callosum, but among these patients, there were also two girls whose mobility was fully dependent on the use of a wheelchair. We can, therefore, confirm the hypothesis that the physiological position and shape of the corpus callosum are associated with better motor skills in patients with MMC.

### 4.3. Hydrocephalus

The term hydrocephalus represents the expansion of the ventricular system of the brain due to the accumulation of cerebrospinal fluid. It occurs in almost all patients with an open form of NTD. The etiology is related to Arnold–Chiari malformation [4].

The relationship between hydrocephalus and CM-II is well established. In the past, physicians and scientists thought that tonsillar herniation was caused by increased intracranial pressure. However, today, we know that the herniation plugged the foramen magnum and caused a disturbance in the flow of CSF. On the basis of this, hydrocephalus arises [4].

The results of the MOMS study showed that prenatal MMC repair reduced the rate of CSF shunt introduction. The need for shunt insertion was 40% in the prenatal surgery group and 82% in the postnatal surgery group. The motor function of patients at 30 months of age significantly improved [16].

Hydrocephalus can worsen the symptoms resulting from the presence of CM-II. Intracranial hypertension due to hydrocephalus should be managed first by performing an endoscopic ventriculostomy of the third cerebral ventricle or by implantation of a cerebrospinal shunt. These solutions take precedence over suboccipital decompression [4].

Half of the patients were diagnosed and treated for CM-II, along with hydrocephalus and myelomeningocele. Patients were indicated for surgery by signs of intracranial hypertension before the age of 12 months. Our study results are similar to the MOMS results. Our preferred method of hydrocephalus treatment was the implantation of a ventriculoperitoneal shunt. In one case, an endoscopic ventriculostomy of the third cerebral ventricle was performed, but even in this patient, a ventriculoperitoneal shunt was later required. 

### 4.4. Functional Lesion Level

In the neurosurgical literature, the functional level of the neural tube defect is related to the need for decompression in the area of the craniocervical junction. The risk of decompression is higher for rostrally located lesions. One of the explanations may be that rostrally located lesions tend to be larger, and they are also associated with significant CSF leakage, which subsequently leads to severe forms of Chiari malformation [12,23].

Table 3 shows that this dependence was not confirmed. Patients who underwent decompression of the craniocervical junction also showed a sacral functional level of defect. In 7/8 (87.5%) cases, the functional level was better than the expected ambulatory status. The functional level was equal to the expected level in only one case.

## 5. Strengths and Limitations 

The strength of this study is its uniqueness for the Slovak population. This is the first work dealing with Chiari II malformation after fetal MMC surgery in the population of Slovak children.

There are limitations to this analysis. The sample size within the prenatal surgery group was not large and limited the ability to detect many associations. The presented group of patients is relatively small but adequate for the size of the population of the Slovak Republic (approximately 5.4 million people) [24]. Bias can be introduced by the retrospective design of the majority of studies. Physicians were not blinded to the surgical group, which could have biased their opinion. Another limitation of the study concerns the radiodiagnostic methods. Prenatal MRI and postnatal MRI examinations were carried out on different devices.

## 6. Future Work

Although our data provide important observations regarding the impact of prenatal repair of the MMC, additional studies are warranted to evaluate the subsequent outcomes. To improve the results of fetal MMC surgery and reduce the risk for the mother and the fetus, it is necessary to continue research. Research should address all aspects of this type of surgery. Continuous follow-ups of the patients in this study are needed to assess the benefits and the effects of prenatal intervention on motor function, mental status, and bowel and bladder continence. Further randomized trials with a uniform design to study the possible complications, advantages, or disadvantages of fetal repair are also essential. Previous cohort studies in the neurosurgical literature have suggested improved outcomes with prenatal surgery for open NTDs. Our future work should focus on how to increase the percentage of independently ambulating patients and reduce the need for shunt implantation in patients with persistent Chiari II malformation and thus achieve favorable psychomotor development. Patients with NTDs have various complications and undergo a large number of operations during their lifetime. Patient care must be multidisciplinary. Future research should focus on the education of health professionals and the patient’s family and on patient care management, with a focus on reducing the need for multiple invasive procedures.

## 7. Conclusions

For NTD patients, surgical treatment is essential. For a long time, the gold standard was a postnatal correction of the defect. Nowadays, fetal surgery is becoming the preferred approach, as it promises a better long-term health status for patients. Our study of eight patients confirms that fetal surgery of MMC reduces the occurrence of Chiari malformations and the associated occurrence of hydrocephalus. The occurrence of a Chiari II malformation in our study was 100% after birth and 87.5% at the age of 12 months. In 12.5% of the cases, the vermis “ascended” postnatally by one segment. A total of 50% of the patients have shunt-dependent hydrocephalus. In this study, a dependence between the functional level of neural tube lesion and the probability of the need for decompression of the craniocervical junction for Chiari malformation was not confirmed. In 87.5% of the cases, the functional level is better than the expected ambulatory status. The functional level is equal to the expected level in only one case.

However, despite the adoption of fetal surgery, the need for decompression of the craniocervical junction is still occasionally preserved.

Despite limitations, the results of this study are especially valuable in prenatal counseling, as they improve the estimate of the probable health status of the child after birth and during their life. This information is essential, and the parents of the fetus need to receive such information during the prenatal consultation when they are deciding whether to continue or terminate the pregnancy.

## Figures and Tables

**Table 1:** Overview of the condition of individual patients from the cohort and the presence of Chiari II malformation on their prenatal and postnatal MR images.

Patient	Sex	Presence of Chiari II Malformation on Prenatal MRI	Presence of Chiari II Malformation on Postnatal MRI After 12 Months	The Need for Decompression of the CCJ for Clinical Difficulties	The Need for ETV/CSF Shunt
1	Male	+	−	−	−
2	Female	+	+	+	−
3	Male	+	+	−	−
4	Male	+	+	+	+
5	Female	+	+	−	+
6	Female	+	+	−	−
7	Female	+	+	−	+
8	Female	+	+	−	+

**Table 2:** Status of the corpus callosum of each patient from the cohort according to postnatal MRI and ambulatory status of our patients.

Patient #	Birth Year	Corpus Callosum Morphology	Current State of Ambulatory Status in 2024	Anatomical Level of Lesion on MRI
1	2016	Normal form	Independent walking with supporting braces	T12-L5/6
2	2017	Dysgenetic	Independent walking with supporting braces	L5
3	2018	Normal form	Stands and walks with the help of another person	T12-S5
4	2018	Dysgenetic	He walks on uneven terrain and upstairs with the help of another person; otherwise, he walks independently	L4-S2
5	2018	Dysgenetic	Mobile in a wheelchair	T12-S2
6	2019	Dysgenetic	Stands and walks with the help of another person	L2-L3
7	2019	Normal form	Stands and walks with the help of another person	L2-L3
8	2020	Dysgenetic	Mobile in a wheelchair	T11-L1

**Table 3:** Dependence between the functional level of the neural tube lesion and the probability of the need for decompression of CCJ for Chiari malformations.

Functional Lesion Level	Number of Patients with a Lesion in the Area	Number of Patients Who Underwent Decompression of CCJ
Thoracic	1	0
High-lumbar	0	0
Mid-lumbar	2	0
Low-lumbar	3	0
Sacral	2	2

## Data Availability

Data are contained within the article.
